# Life-Threatening Influenza, Hemophagocytic Lymphohistiocytosis and Probable Vaccine-Strain Varicella in a Novel Case of Homozygous *STAT2* Deficiency

**DOI:** 10.3389/fimmu.2020.624415

**Published:** 2021-02-18

**Authors:** Bishara J. Freij, Aidan T. Hanrath, Rui Chen, Sophie Hambleton, Christopher J. A. Duncan

**Affiliations:** ^1^ Pediatric Department, Beaumont Children's Hospital, Royal Oak, MI, United States; ^2^ Oakland University William Beaumont School of Medicine, Rochester, MI, United States; ^3^ Immunity and Inflammation Theme, Translational and Clinical Research Institute, Newcastle upon Tyne, United Kingdom; ^4^ Great North Children's Hospital, Newcastle upon Tyne Hospitals NHS Foundation Trust, Newcastle upon Tyne, United Kingdom; ^5^ Royal Victoria Infirmary, Newcastle upon Tyne Hospitals NHS Foundation Trust, Newcastle upon Tyne, United Kingdom

**Keywords:** type I interferon, type I and type III interferon signaling, JAK-STAT signaling pathway, viral disease, hyperinflammation, interferon stimulated gene, inborn errors of antiviral immunity, varicella zoster virus

## Abstract

STAT2 is a transcription factor that plays an essential role in antiviral immunity by mediating the activity of type I and III interferons (IFN-I and IFN-III). It also has a recently established function in the negative regulation of IFN-I signaling. Homozygous STAT2 deficiency is an ultra-rare inborn error of immunity which provides unique insight into the pathologic consequence of STAT2 dysfunction. We report here a novel genetic cause of homozygous STAT2 deficiency with several notable clinical features. The proband presented aged 12 months with hemophagocytic lymphohistiocytosis (HLH) closely followed by clinical varicella, both occurring within three weeks of measles, mumps, and rubella (MMR) and varicella vaccinations. There was a history of life-threatening influenza A virus (IAV) disease 2 months previously. Genetic investigation uncovered homozygosity for a novel nonsense variant in *STAT2* (c. 1999C>T, p. Arg667Ter) that abrogated STAT2 protein expression. Compatible with STAT2 deficiency, dermal fibroblasts from the child demonstrated a defect of interferon-stimulated gene expression and a failure to mount an antiviral state in response to treatment with IFN-I, a phenotype that was rescued by lentiviral complementation by wild type *STAT2*. This case significantly expands the phenotypic spectrum of STAT2 deficiency. The occurrence of life-threatening influenza, which has not previously been reported in this condition, adds *STAT2* to the list of monogenetic causes of this phenotype and underscores the critical importance of IFN-I and IFN-III to influenza immunity. The development of probable vaccine-strain varicella is also a novel occurrence in STAT2 deficiency, implying a role for IFN-I/III immunity in control of attenuated varicella zoster virus *in vivo* and reinforcing the susceptibility to pathologic effects of live-attenuated viral vaccines in disorders of IFN-I immunity. Finally, the occurrence of HLH in this case reinforces emerging links to hyperinflammation in patients with STAT2 deficiency and other related defects of IFN-I signaling—highlighting an important avenue for further scientific enquiry.

## Introduction

Inborn errors of antiviral immunity are a rich source of insight into essential pathways of host defence against specific viral pathogens. In addition to teaching us fundamental but clinically relevant lessons, these ultra-rare disorders also inform the development of novel therapeutics and vaccines for viral disease. Discoveries in this field have shed light on the critical role played by innate type I and type III interferons (IFN-I and IFN-III) in human antiviral immunity [reviewed in (1, 2)]. The importance of these pathways was highlighted recently by the discovery of defects of IFN-I immunity in patients with life-threatening SARS-CoV-2 infection (3, 4).

IFN-I, comprising 13 subtypes of IFNα, alongside IFNβ, IFNε, IFNκ and IFNω, was discovered more than 60 years ago as the mediator of ‘viral interference’ (5). The discovery of IFN-III (IFNλ1-IFNλ4) followed more recently (6–8). IFN-I signals through a single ubiquitously expressed receptor, a heterodimer of IFNAR1 and IFNAR2. By contrast, expression of the receptor for IFN-III—composed of IFNL1 and IL10RB—is more confined to mucosal surfaces and some immune cell subsets (9). Ligation of IFNAR or IFNLR activates an ostensibly similar downstream JAK-STAT signaling cascade culminating in the activation of two major transcription factor complexes—interferon stimulated gene factor 3 (ISGF3, composed of tyrosine phosphorylated STAT1, STAT2, and IRF9) and the gamma activated factor (GAF, homodimers of pSTAT1).

To date, genetic lesions have been identified in all major components of the canonical IFN-I and IFN-III signaling pathways (1). These disorders are recognized clinically by a phenotype of isolated vulnerability to severe and/or recurrent viral disease (with the exception of complete STAT1 deficiency where the pathogen susceptibility profile is broader, encompassing mycobacterial disease). STAT2 deficiency was among the first of these disorders to be discovered, and is characterized by susceptibility to both live-attenuated viral vaccines and naturally acquired viral pathogens (10–12), presumably reflecting the combined impact on systemic IFN-I and mucosal IFN-III responses.

STAT2 is a ubiquitously expressed transcription factor. Unlike other STAT family members, which are relatively promiscuous, STAT2 participates in a narrower range of cytokine signaling pathways (IFN-I and IFN-III only). It is frequently targeted by pathogenic viruses as a means of subverting IFN restriction (13). Recent findings indicate that STAT2 also plays an important role in immunoregulation, by supporting the function of USP18 (14) – a key negative regulator of IFN-I signaling (15–18)—and also by sequestering STAT1 in the cytosol (19). Isolated failure of the regulatory function of STAT2 towards USP18 underlies a severe sterile autoinflammatory disease mediated by persistent, uncontrolled IFN-I signaling (20, 21).

Hyperinflammation is also increasingly recognized in a minority of STAT2-deficient patients (11), although the underlying pathomechanism is not known and consequently there is limited information about how to approach its management. Severe disease meeting clinical criteria for hemophagocytic lymphohistiocytosis (HLH) remains unusual in STAT2 deficiency, having been reported in only a single individual to date (12).

In addition to defining organizing principles of IFNs in antiviral immunity, monogenic disorders also provide information about key aspects of the host response to individual medically important viral pathogens, for example influenza (22). Susceptibility to life-threatening influenza in otherwise healthy individuals is an emerging clinical phenotype associated with several inborn errors of IFN immunity, including homozygous loss of function mutations in *IRF7*, *IRF9*, and *TLR3* (23–25). Defects in *STAT2* have not hitherto been associated with this phenotype.

In this report we describe a new case of complete STAT2 deficiency with several novel features, expanding the spectrum of disease and thereby informing understanding of the broader roles of IFN-I/III signaling in human immunity.

## Materials and Methods

### Ethics Statement and Consent

This study was performed in accordance with the principles of the Helsinki declaration. Written parental consent was obtained for genetic testing. Ethical approval for studies on patient fibroblasts was granted by the NRES Committee North East - Newcastle & North Tyneside 1 (Ref: 16/NE/0002).

### Whole Exome Sequencing

Whole exome sequencing of DNA extracted from whole blood of the proband was performed commercially on the Illumina platform by Invitae Corporation (California, USA). Alignment and variant analysis were undertaken according to current Genome Analysis Toolkit (GATK) joint calling best practice guidelines using the reference assembly GRCh37 (hg 19) for alignment. Carriage of the *STAT2* variant by the proband, and all family members, was tested by Sanger sequencing of DNA isolated from whole blood by local diagnostic genetic laboratories according to standard methodologies.

### *In Silico* Prediction Tools

PhastCons is a method to determine the grade of conservation of a given nucleotide, given as a score from 0 to 1 (26). MutationTaster uses values which are precomputed and offered by UCSC (27). The Combined Annotation Dependent Depletion (CADD) score is a tool for integrating conservation and deleteriousness predictions (28).

### Cells, Cytokines, Immunoblotting

Dermal fibroblasts from patient II:3 and three healthy controls were obtained by standard methods and cultured in Dulbecco’s Modified Eagle’s Medium supplemented by 10% foetal calf serum and 1% penicillin/streptomycin (DMEM-10). Human recombinant IFNα2b (Intron-A, Schering-Plough, USA) and IFNγ (Immunikin, Boehringer Ingelheim, Germany) were used at 1000 IU/ml. Immunoblotting was carried out as previously described (20). Antibodies used are described in **Table 1**.

**Table 1:** Antibodies.

Antibody	Host	Dilution	Source	Code
STAT2 N-term	Mouse	1:2000	SCB	sc-1668
STAT2 C-term	Rabbit	1:2000	SCB	sc-476
pSTAT2	Rabbit	1:2000	CST	8841
STAT1	Rabbit	1:1000	CST	9172
pSTAT1	Rabbit	1:1000	CST	7649
MX1/2/3	Rabbit	1:1000	SCB	sc-50509
IFIT1 (ISG56)	Goat	1:500	SCB	sc-82946
RSAD2	Rabbit	1:1000	CST	13996
α-tubulin	Mouse	1:10,000	CST	3873
Anti-rabbit HRP-conjugated	Goat	Various	CST	7074
Anti-mouse HRP-conjugated	Horse	Various	CST	7076
Anti-goat HRP-conjugated	Rabbit	1:4000	Millipore	401515

CST, Cell Signaling Technologies; SCB, Santa-Cruz Biotechnology.

### Viral Cytopathic Effect Reduction Bioassay

Monolayers of dermal fibroblasts were infected with encephalomyocarditis virus (EMCV, kindly provided by R. E. Randall and D. Young, St. Andrew’s University, UK) at 10^5^ pfu/ml for 24 h before fixation with 4% formaldehyde and staining with 0.5% crystal violet. Plates were washed extensively then allowed to air dry. The remaining cell membrane-bound stain was solubilized with methanol and absorbance at 595 nm measured on a TECAN Sunrise plate reader (Tecan, Switzerland). Subsequent analysis was undertaken as previously described (20).

### Lentiviral Complementation

The bicistronic lentivirus vector (pHR-SIN-CSGW) containing full-length WT human *STAT2*, or a control vector expressing GFP, was used (kindly provided by R.E. Randall, St Andrew’s University, UK). Lentiviruses were produced by co-transfection of psPAX2, pCMV-VSV-G and lentiviral transfer plasmid in HEK293T cells using PEI (Sigma-Aldrich, Gillingham, UK) or FuGene (Promega, Wisconsin, USA). Virus containing supernatants were harvested at 48 h post-transfection, filtered (0.45 μm sterile filter) and concentrated 100-fold with Lenti-X™ Concentrator (TaKaRa, Shiga, Japan) according to manufacturer’s instructions. Cells were spinoculated in six-well plates (1.5 h, 2000 rpm), with target or null control viral particles, at various dilutions in a total volume of 0.5 ml DMEM-10 containing hexadimethrine bromide (Polybrene, 8 μg/ml, Sigma-Aldrich). Cells were rested in virus-containing medium for 8 h then incubated in fresh DMEM-10 until 48 h, when they were subjected to selection with 2.0 μg/ml puromycin (Sigma-Aldrich). Antibiotic-containing medium was refreshed every 72 h.

### Statistical Analysis

Continuous data from the EMCV CPE bioassay were normalized prior to parametric tests of significance (ANOVA with Sidak’s post-test correction for multiple comparisons). Analysis was undertaken using GraphPad Prism version 8.0, with (corrected) two-tailed alpha < 0.05 the threshold for significance.

## Results

### Case Summary

We investigated a 12-month-old girl who presented with high fever, diarrhea, new onset vomiting, nasal congestion, cough, irritability, and lethargy. Six days before onset of this illness, she had been inoculated with age-appropriate vaccines (MMR, varicella zoster virus, hepatitis A, *Haemophilus influenzae* type b, and the 13-valent pneumococcal conjugate vaccines) despite nasal congestion and clear rhinorrhea. The child was born to distantly related but healthy Honduran parents at 38 weeks of gestation. Her own medical history was remarkable for significant viral illness: at 9 months of age, she had a prolonged febrile illness with seizures, triggered by seasonal coronavirus HKU1 infection. At 10 months of age, she experienced severe influenza A pneumonia with very high fever and progressive respiratory failure despite treatment with oseltamivir, ceftriaxone, methylprednisolone and oxygen delivered by high-flow nasal cannulae. She required intubation and mechanical ventilation for 3 days but recovered.

On admission, her physical examination only showed nasal congestion. The laboratory parameters were largely unremarkable (**Table 2**). A partial septic screen yielded >100,000 colony forming units/ml of *Escherichia coli* from urine and a positive PCR for rhinovirus/enterovirus RNA from a nasopharyngeal swab sample. Blood cultures collected on days 4 and 6 and were sterile. High fever continued despite intravenous ceftriaxone for presumed pyelonephritis. She was on the Paediatric ward for 2 days with continuing high fever up to 41°C. On day 3, she was transferred to the Paediatric Intensive Care Unit (PICU) because of poor bilateral lower extremity perfusion that failed to improve on multiple fluid boluses. This was accompanied by persistent tachycardia, increased work of breathing, and a diffuse erythematous rash. Extensive imaging revealed no evidence of an infective focus in the renal tract (ultrasonography), chest (repeated plain x-radiography), abdomen/pelvis (contrast CT) or heart (echocardiography). Virology investigations were consistent with human herpes virus type 6 (HHV6) reactivation, based on positive blood PCR (23,500 (log 4.4) copies/ml of HHV-6 type B), positive HHV6 IgG at 1:80 (negative if <1:10) and negative IgM (<1:20) on day 6. Testing was not undertaken for MMR viruses; samples were not stored to enable retrospective testing. Persistent high fever was accompanied by worsening cytopenias, elevated triglycerides, elevated ferritin to ≥500 ng/ml, and elevated soluble interleukin-2 receptor level (**Table 2**). These results met 5 of 8 criteria for the diagnosis of hemophagocytic lymphohistiocytosis (HLH) (13). An attempt at bone marrow aspiration on day 9 of admission was aborted due to apnea and profound desaturation. She was quickly resuscitated and returned to baseline within a few hours while hematologic parameters began spontaneously improving by day 10. Fever abated after 15 days and the child was discharged on no antibiotics. Treatment with corticosteroids and/or other immunomodulators was planned after the bone marrow aspiration, but not administered because of clinical and laboratory evidence suggestive of spontaneous HLH resolution on day 10. The diagnosis was considered to be transient HLH triggered by HHV6 infection.

**Table 2:** Laboratory data by day of fever.

Laboratory data	d4	d6	d7	d8	d9	d10	d11	d12	d15	Normal range
Hemoglobin (g/dL)	12.1	10.4	10.1	9.4	9.7	9.8	8.9	8.1	8.9	10.2–14.7
Hematocrit (%)	34.4	30.1	29.2	26.9	27	26.3	25.7	24.5	27.3	30.8–43.7
White blood cell count (10^9^/L)	7.6	3.5	2.3	1.4	1.5	2.7	3.5	5.6	9.1	6.0–17.5
Absolute neutrophil count (10^9^/L)	4	2.2	1.2	0.8	0.8	1.1	1.4	2.4	3.1	1.5–8.5
Absolute lymphocyte count (10^9^/L)	2.3	1	1	0.6	0.7	1.3	1.7	2.6	4.6	4.0–10.5
Absolute monocyte count (10^9^/L)	1.2	0.2	0.1	9.1	0	0.2	0.3	0.4	0.7	0.3–0.9
Platelet count (10^9^/L)	242	144	127	107	85	85	101	135	534	150–450
AST (U/L)	66	57			221	125	104	82	73	10.0–37.0
ALT (U/L)	57	38			100	94	85	78	55	5.0–30.0
C-reactive protein (mg/L)		76								0.0–8.0
Ferritin (ng/ml)					1980					12–207
Lactate dehydrogenase (U/L)					1043					190–420
Triglycerides (mg/dL)					289					30–120
D-dimer (ng/ml)						>9,999				0–499
Fibrinogen (mg/dL)						71	65			175-375
Soluble interleukin-2 receptor (pg/ml)						14740				<1034

These abnormalities resolved on retesting in convalescence.

Immediately after hospital discharge she developed a diffuse vesicular rash from which varicella-zoster virus (VZV) DNA was detected by PCR. This occurred 20 days after receiving the varicella vaccine. She was treated with acyclovir for 5 days (20 mg/kg every 6 h) without complication. There was no attempt to ascertain by molecular testing whether VZV was the vaccine strain. However the child had no exposure to individuals with chickenpox or shingles at home or in hospital. Based on molecular typing of rash-associated virus in post-marketing varicella vaccine surveillance in the US and Europe, the timing of this rash is more consistent with vaccine-strain varicella (median onset 17–21 days post-vaccination) than with wild type varicella (median interval: 7 - 8 days post-vaccination) (29, 30). Thus on a balance of probabilities, our patient likely experienced dissemination of vaccine-strain VZV rather than wild type varicella.

At 14 months of age, immunological work up was undertaken which revealed a polyclonal increase in IgG, protective antibody levels against measles, VZV, diphtheria, and tetanus and normal lymphocyte subset analysis (**Table 3**). Subsequently the proband has experienced multiple illnesses secondary to rhinovirus, parainfluenza virus type 3, and human metapneumovirus (**Table 4**). Now 3 years of age, her physical growth and developmental milestones have been normal. Further live vaccines have been withheld. Prophylactic immunoglobulin therapy was not felt to be indicated.

**Table 3:** Immunophenotyping results, patient II:3.

Parameter	Value	Normal range
Total lymphocytes	3,600	1600–6,700
CD3+ CD4+	1,008	1,000–4,600
CD3+ CD8+	756	400–2,100
CD3 CD19+	1,368	600–2,700
CD3− CD56+	216	200–1,200
IgG (mg/dL)	1,170	300–900
IgG1 (mg/dL)	979	160–562
IgG2 (mg/dL)	218	24–98
IgG3 (mg/dL)	90	17–64
IgG4 (mg/dL)	201	0–22
IgM (mg/dL)	63	15–70
IgA (mg/dL)	40	40–160
IgE (IU/ml)	6	0.62–1.6
Tetanus IgG (IU/ml)	4.5	> 0.1
Diphtheria IgG (IU/ml)	0.8	> 0.1
Measles IgG (AU/ml)	> 300	≥ 30
VZV IgG (Index units)	1033	≥ 135

**Table 4:** Summary of infection history in patient II:3.

Age	Illness
9 months	Prolonged fever with seizure; coronavirus HKU1 (non-COVID-19) infection
10 months	Influenza A (H3) pneumonia, mechanical ventilation for 3 days
12–13 months	HLH with human herpes virus type 6B replication, and varicella, 20 days after Varivax, MMR, hepatitis A, *Haemophilus influenzae* type b, and 13-valent conjugate pneumococcal vaccines. Resolved spontaneously.
14 months	Pulmonary consolidation and fever for 4 days associated with rhinovirus/enterovirus
15 months	Rhinovirus/enterovirus upper respiratory tract infection with fever for 7 days
17 months	Suppurative otitis media with fever
24 months	Febrile parainfluenza virus type 3 upper respiratory tract infection
25 months	Mixed upper respiratory tract infection with parainfluenza virus type 3, human metapneumovirus, and rhinovirus/enterovirus
26 months	Upper respiratory tract infection with rhinovirus/enterovirus, with 5 days of fever up to 39.3^o^C and development of lower extremity papular and urticarial rash

### *STAT2* Variant Identification

**Figure 1:**
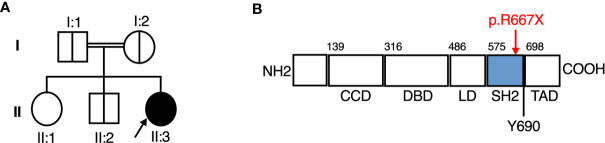
A novel Arg677Ter variant causing autosomal recessive *STAT2* deficiency. **(A)** Family pedigree. **(B)** STAT2 protein domains, showing the Arg677Ter variant.

Suspecting a genetic aetiology in this case, whole exome sequence analysis and addition/deletion testing of 207 genes associated with primary immunodeficiency was undertaken on whole blood DNA from the proband, identifying a homozygous predicted pathogenic variant in *STAT2* (c.1999C>T [p.Arg667Ter]) and a heterozygous variant in *DOCK2* [c.54-1G>T], the latter of unlikely clinical relevance. Sanger sequencing analysis confirmed the presence of *STAT2* c.1999C>T in the homozygous state in the proband. This variant was heterozygous in both parents and a clinically unaffected sibling, consistent with segregation of an autosomal recessive trait (**Figure 1A**).

### The Arg667Ter Mutation Leads to Complete STAT2 Deficiency

**Figure 2:**
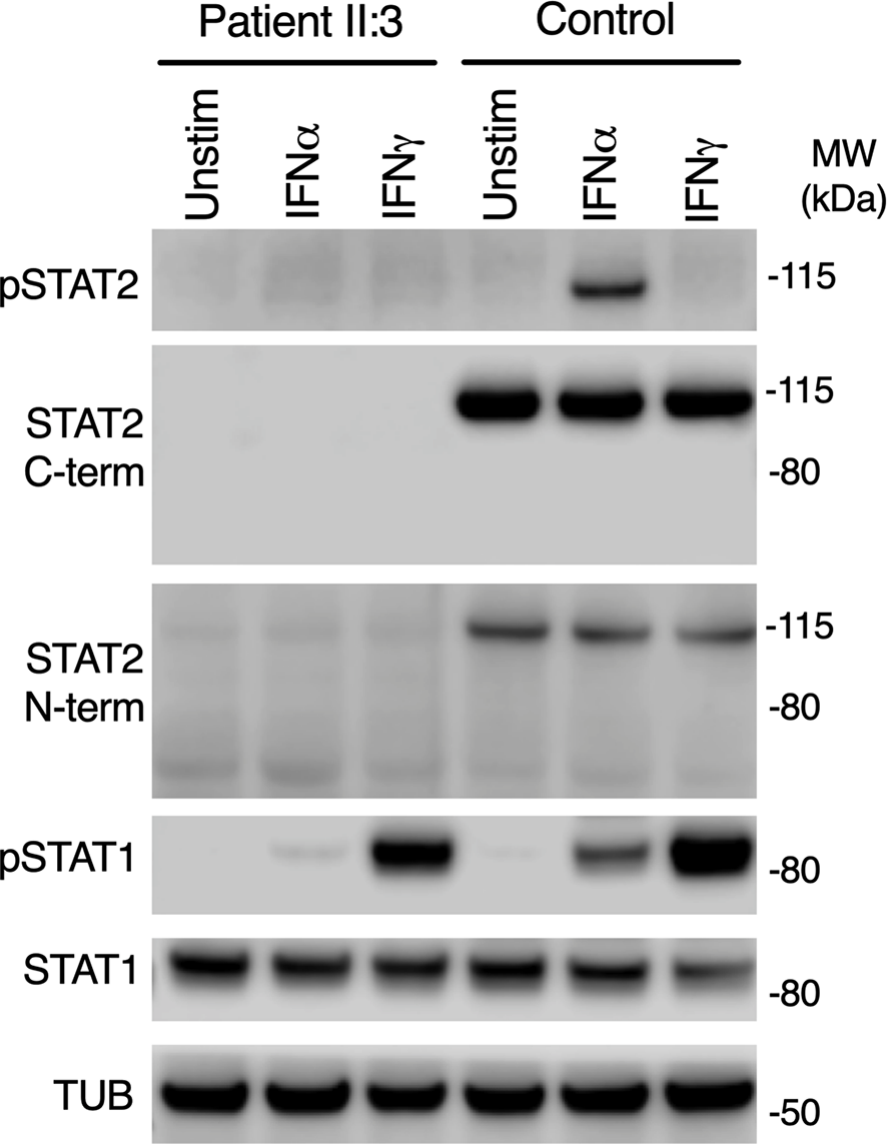
Impaired type I but preserved type II IFN signaling in patient cells. Immunoblot of whole-cell lysates prepared from primary dermal fibroblasts from patient II:3 or a healthy control treated with 1000 IU/ml of IFNα2b or IFNγ for 30 mins, showing absence of STAT2 using N-terminal and C-terminal antibodies. Signaling to IFNγ was preserved. Representative of n=3 repeat experiments.

*STAT2* c.1999C>T variant was absent from databases of genomic variation (gnomAD) (31). The variant introduced a premature stop codon in place of a conserved arginine residue at position 667 (p.Arg667Ter) in the SH2 domain of STAT2 protein (**Figure 1B**), which was predicted to be deleterious by *in silico* tools PhastCons (0.94, max 1.0), MutationTaster and CADD (36, max 36). To assess STAT2 protein expression, lysates were prepared from dermal fibroblasts obtained from the child. No truncated protein product was identified when probing primary dermal fibroblast lysates with antibodies raised against either N-terminal or C-terminal STAT2 antigens (**Figure 2**), indicative of complete STAT2 deficiency.

### Defective IFNAR But Preserved IFNGR Signaling

STAT2 is activated by tyrosine phosphorylation downstream of the receptors for IFN-I and IFN-III, but not type II IFN (IFNγ), whereas STAT1 is activated by all IFN subtypes. We treated primary dermal fibroblasts from the child alongside healthy controls with recombinant IFNα2b or IFNγ. Probing whole cell lysates for tyrosine phosphorylated STAT2 and STAT1 proteins confirmed normal phosphorylation of STAT1 in response to IFNγ in both patient and controls, implying that IFNGR signaling was intact in patient cells (**Figure 2**). STAT1 was also phosphorylated in patient lysates in response to IFNα2b at this early time point, although the abundance was reduced, as reported in STAT2-deficient cell-lines (19).

### Impaired ISG Expression and Induction of the Antiviral State

**Figure 3:**
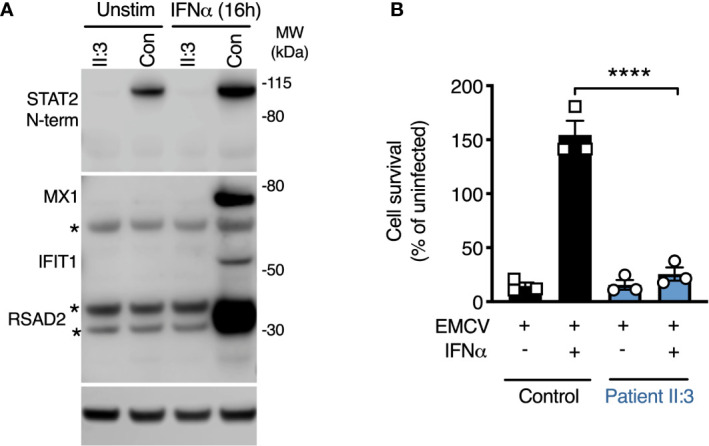
Defective induction of the type I IFN dependent antiviral state. **(A)** Immunoblot analysis of whole cell lysates prepared from patient II:3 and control fibroblasts following overnight treatment with IFNα2b (1000 IU/ml) demonstrating the failure to induce the ISG products of *MX1*, *RSAD2*, and *IFIT1* in patient fibroblasts. Stars represent non-specific bands. Representative of n=3 repeat experiments. **(B)** Encephalomyocarditis virus (EMCV) cytopathic effect reduction bioassay demonstrating the failure of IFNα2b pre-treatment (1000 IU/ml) to induce the antiviral state in patient but not control fibroblasts. Displayed are mean ± SEM of n=3 repeat experiments ****P<0.001 ANOVA with Sidak’s post-test.

The predicted consequence of STAT2 deficiency is to compromise the assembly of a functional ISGF3 complex, thus impairing the expression of interferon-stimulated genes (ISGs). Consistent with this prediction, the expression of several ISGF3-dependent ISG proteins—MXA, IFIT1, and RSAD2 was absent in whole cell lysates prepared from STAT2-deficient patient fibroblasts exposed to IFNα2b overnight (**Figure 3A**). To address the functional consequence of impaired ISG induction, we infected cells with encephalomyocarditis virus (EMCV), a picornavirus cytopathic for human cells in the absence of exogenous IFNs. In this experiment, fibroblasts were pre-treated with IFNα2b overnight at a dose that was previously determined to prevent cytopathic effect (CPE) in control cells, then examined at 24 h post infection. STAT2-deficient cells were susceptible to CPE irrespective of IFNα2b exposure, confirming that induction of the antiviral state was compromised (**Figure 3B**).

### STAT2 Complementation Restored Functional IFN-I Responses

**Figure 4:**
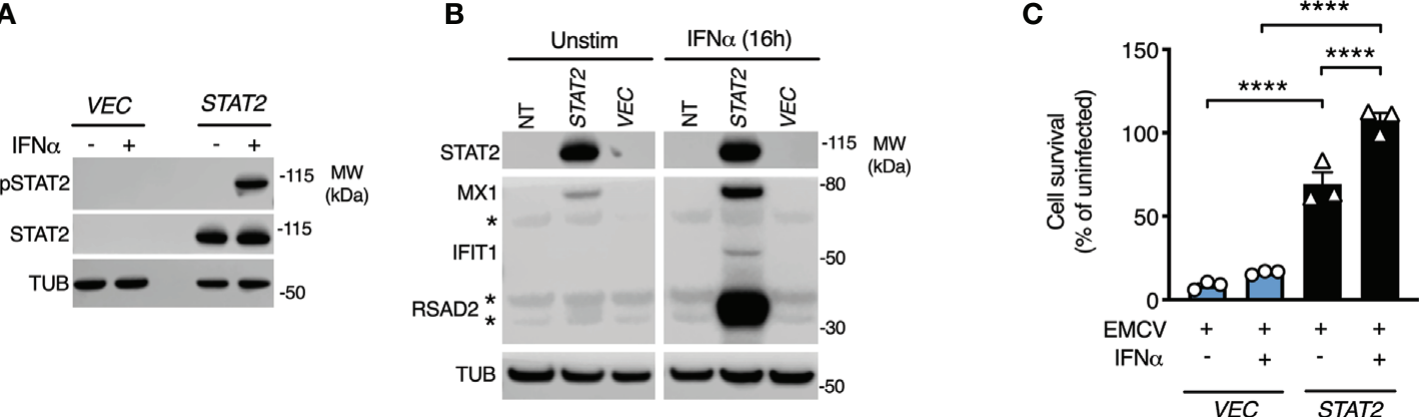
STAT2 complementation rescues the defect of type I IFN signaling. Fibroblasts from patient II:3 were transduced by lentiviruses expressing either empty vector (*GFP*) or *STAT2*. Immunoblot analysis of whole cell lysates prepared from transduced fibroblasts demonstrating **(A)** restoration of STAT2 protein expression and tyrosine phosphorylation in response to 30 min IFNα2b 1000 IU/ml and **(B)** restoration of ISG expression in response to 16h treatment with IFNα2b (1000 IU/ml) in STAT2 complemented patient fibroblasts. **(C)** EMCV cytopathic effect reduction bioassay demonstrating the failure of IFNα2b pre-treatment (1000 IU/ml) to induce the antiviral state in GFP complemented but not STAT2- complemented II:3 fibroblasts. Displayed are mean ± SEM of n=3 repeat experiments ****P<0.001 ANOVA with Sidak’s post-test.

To prove definitively that the loss of STAT2 protein was causal, we complemented patient cells with full-length human *STAT2* delivered by lentiviral transduction (10). Complemented cells were compared to patient fibroblasts transduced in parallel with a control lentiviral vector expressing *GFP*. Lentiviral transduction of *STAT2* in patient fibroblasts restored STAT2 protein expression and its phosphorylation in response to IFNα2b treatment (**Figure 4A**). This translated into the restoration of both ISG induction and formation of a cellular antiviral state in response to IFNα2b (**Figures 4B, C** **)**, thereby confirming the genotype-phenotype association. *STAT2* overexpression led to the induction of MX1 and a partial antiviral state in the absence of IFNα2b exposure, as previously observed (10).

## Discussion

This case informs understanding of the function of STAT2 in both antiviral immunity and immunoregulation. STAT2 is unique among human STATs, by virtue of both its narrow spectrum of activity (within IFN-I/III pathways) and its direct participation in both positive and negative regulation. Defining the molecular and clinical consequences of *STAT2* variants provides not only clinically relevant information, but also fundamental insight into the involvement of STAT2, and IFN-I/III more generally, in human immunity.

Our data identify homozygous STAT2 deficiency as the fourth inborn error of IFN immunity underpinning severe influenza A virus (IAV) (23–25). Life-threatening IAV has been previously associated with inborn errors of IFN-I/III production due to homozygous loss of function defects in *IRF7* (23) and more recently *TLR3* (25) (previously recognized to underlie susceptibility to herpes encephalitis (32)). Whilst these disorders suggested that IFN-I/III signaling forms a critical aspect of the initial response to IAV infection, it was not possible to exclude the additional contribution of IFN-independent responses mediated by pattern-recognition receptor signaling. However, it is now clear that IFN-I/III signaling protects against severe manifestations of IAV - based on the recent report of life-threatening IAV in a patient with a homozygous loss of function variant in *IRF9* (24), together with this report in STAT2 deficiency. It should be noted that severe IAV displays incomplete clinical penetrance in patients with mutated *TLR3*, *IRF7*, *IRF9*, or *STAT2*. This is not unexpected, since in the context of defects of pathogen defence there are multiple factors that likely contribute to variable expressivity, including: variable pathogen exposure, infecting dose, prior vaccination and compensation by adaptive immunity.

All of the molecular defects hitherto associated with life threatening IAV impact the synthesis of, or transcriptional response to, both IFN-I and IFN-III. The relative contribution of IFN-I and IFN-III systems to IAV defence in humans remains an open question. A phenotype of vulnerability to severe IAV has not been reported to date in selective defects of either IFN-I signaling [i.e. IFNAR1 (33, 34) or IFNAR2 (35) deficiency], nor IFN-III signaling [i.e. IL10RB deficiency (36)], suggesting that neither pathway in isolation is essential. Whilst this could be cautiously interpreted as evidence of functional redundancy, an important caveat is that only a handful of cases of IFNAR deficiency have been identified to date, and a broader phenotype might emerge as more cases are discovered and/or followed up. However this assessment has some support from studies of *Ifnar1* ^−/−^ and *Ifnlr* ^−/−^ mice, which suggest that IFN-I and IFN-III are effectively capable of functional compensation against severe disease due to influenza: only when both systems are simultaneously disabled, in *Ifnar1* ^−/−^ *Ifnlr1* ^−/−^ double knockout mice, did mice consistently succumb to disease (37). However more recent data indicate that the IFN-III system plays a nonredundant role in protection of the upper airway, and in limiting transmission of IAV in mice (38), suggesting a distinct function for IFN-III. On this point it is also very relevant that specific defects of IFNAR signaling have recently been implicated in patients with life threatening SARS-CoV-2 infection, another pathogen naturally acquired via the respiratory tract (3). It is clear that SARS-CoV-2 has a broader cellular tropism and causes pathology in a wider range of tissues than IAV. Considering the mucosa-restricted expression of IFNLR compared to IFNAR, this broader tropism of SARS-CoV-2 might explain the apparently greater reliance of the host on IFN-I for protection. Nevertheless, specific mechanistic studies are required to determine the relative functional importance of IFN-I and IFN-III at the mucosa in human systems.

Disease caused by live-attenuated parenteral viral vaccines continues to serve as a clear signal of compromised IFNAR signaling (10–12, 34, 35). Another novel observation in this case is the occurrence of clinical varicella following VZV immunization, which has not previously been described in STAT2 deficiency. The implication is that vaccine-strain VZV may, like other live-attenuated vaccines such as MMR, be effectively controlled by innate IFNs *in vivo* (39), consistent with a previous report of VZV dissemination in a child with IRF9 deficiency (40). Regrettably in our case, molecular analysis was not undertaken at the time of varicella diagnosis to definitively prove the vaccine origin of VZV, nor was material stored to enable retrospective analysis. It is also notable that dissemination of MMR virus was not identified in this case, despite a suggestive temporal association between administration of MMR and the onset of clinical disease. Most individuals with molecular defects of IFN-I immunity that are known to have received MMR have developed pathologic dissemination. There are however exceptions. These include a patient with IFNAR1 deficiency who experienced no apparent illness following MMR, but nevertheless subsequently developed severe systemic disease due to dissemination of live-attenuated yellow fever vaccine (34). Another IFNAR1 deficient patient developed a self-limiting febrile illness following MMR, whereas her cousin died from complications of MMR (33). Thus, MMR dissemination is strongly associated with defects of IFN-I/III immunity, but is not inevitable. Again, it should be noted that in our case, blood and/or mucosal samples were not analyzed by PCR for MMR viral detection, and no clinical material was available to retrospectively test. Therefore occult MMR viral replication cannot be excluded as a potential contribution to HLH.

The occurrence of HLH is also unusual in STAT2 deficiency but underlines an emerging phenotype of immune dysregulation. Of the nine STAT2-deficient patients reported to date, three developed overt inflammatory manifestations, including prolonged febrile illness responsive to immunoglobulin therapy in two (11) and illness meeting HLH criteria in one (12). The pathomechanism accounting for hyperinflammation in STAT2 deficiency remains uncertain and represents an important avenue for future research. Interestingly, inflammatory disease has also been reported in other molecular defects of IFN-I/IFN-III signaling, including IFNAR2, STAT1 and IRF9 deficiency (12, 35, 40, 41). In most cases, hyperinflammation arose in the context of intercurrent viral infection, suggesting that viral infection might act as a trigger. In this regard, there was circumstantial evidence of reactivation of HHV6 in some of these cases (35, 41), as here, but whether this is causal or not remains unclear. However, other factors might also contribute to perpetuating and amplifying the dysregulated inflammatory response, including: (i) delayed control of viral replication (and/or herpesvirus reactivation) leading to a greater antigenic load; (ii) overactivity of compensatory immune pathways, such as IFNγ or IL1β (42, 43), and/or (iii) failure of STAT2-dependent negative feedback upon type I IFN signaling. In the latter case, it is now well established that STAT2 plays an essential role in such regulation by supporting the regulatory function of USP18 (20, 21), and also by restraining IFNAR-independent activity of STAT1 (19). The concurrent loss of the regulatory activity of STAT2 might be an important contributor to these manifestations. From a therapeutic perspective, it remains unclear how best to treat hyperinflammation in STAT2 deficiency. The capacity of IFNγ to compensate for antiviral activity *in vitro* might suggest a potential therapeutic role in controlling viral replication, however this approach may come at a cost of enhancing hyperinflammation. There have been clinical reports of responsiveness to IVIG or steroids (11, 41), although in our case the hyperinflammation resolved without specific treatment. More work is needed to dissect the pathomechanism and inform therapeutic strategies.

In summary, this case significantly expands our clinical understanding of STAT2 deficiency. As the first report of life-threatening IAV in STAT2 deficiency, we can now add *STAT2* to the list of genes implicated in this phenotype – a list that increasingly emphasizes the importance of IFN-I/III (23–25). We also report the first instance of disseminated vaccine-strain VZV in STAT2-deficiency, suggesting a role for innate IFNs in control of live-attenuated VZV vaccines. Finally, as only the second report of HLH in a patient with STAT2-deficiency, this case strengthens the notion that STAT2-deficient patients are vulnerable to hyperinflammation, motivating further investigation of the underlying mechanism(s) and highlighting the enduring capacity of monogenic diseases to uncover novel areas for scientific exploration.

## Data Availability Statement

The raw data supporting the conclusions of this article will be made available by the authors, without undue reservation.

## Ethics Statement

The studies involving human participants were reviewed and approved by NRES Committee North East—Newcastle & North Tyneside 1 (Ref: 16/NE/0002). Written informed consent to participate in this study was provided by the participants’ legal guardian/next of kin.

## Author Contributions

Clinical care: BF. Conception: BF, SH, CD. Experimental work: AH, RC, CD. Data analysis: AH, RC, CD. Funding acquisition: SH, CD. Manuscript writing: BF, CD. Manuscript reviewing and editing: BF, AH, RC, SH, CD. All authors contributed to the article and approved the submitted version.

## Funding

SH and CD are funded by the Wellcome Trust (207556/Z/17/Z and 211153/Z/18/Z respectively). AH and CD received additional grant funding from the British Medical Association.

## Conflict of Interest

The authors declare that the research was conducted in the absence of any commercial or financial relationships that could be construed as a potential conflict of interest.
